# The role of population change in the increased economic differences in mortality: a study of premature death from all causes and major groups of causes of death in Spain, 1980–2010

**DOI:** 10.1186/s12889-015-1678-5

**Published:** 2015-04-02

**Authors:** David Martínez, Carolina Giráldez-García, Estrella Miqueleiz, María E Calle, Juana M Santos, Enrique Regidor

**Affiliations:** Department of Preventive Medicine and Public Health, Faculty of Medicine, Universidad Complutense de Madrid, Madrid, Spain; Instituto de Investigación Sanitaria del Hospital Clínico San Carlos (IdISSC), Madrid, Spain; Departament of Sociology, Universidad Pública de Navarra, Pamplona, Spain; CIBER Epidemiología y Salud Pública (CIBERESP), Madrid, Spain

**Keywords:** Population change, Per capita income, Mortality, Causes of death, Spain

## Abstract

**Background:**

An increase has been observed in differences in mortality between the richest and poorest areas of rich countries. This study assesses whether one of the proposed explanations, i.e., population change, might be responsible for this increase in Spain.

**Methods:**

Observational study based on average income, population change and mortality at provincial level. The premature mortality rate (ages 0–74 years) was estimated for all causes and for cancer, cardiovascular disease and external causes across the period 1980–2010. In the years analysed, provinces were grouped into tertiles based on provincial income, with the mortality rate ratio (MMR) being estimated by taking the tertile of highest-income provinces as reference. Population change was then controlled for to ascertain whether it would modify the rate ratio.

**Results:**

In all-cause mortality, the magnitude of the MRR for provinces in the poorest versus the richest tertile was 1.01 in 1980 and 1.12 in 2010; in cardiovascular mortality, the MMRs for these same years were 1.08 and 1.31 respectively; and in the case of cancer and external-cause mortality, MMR magnitude was similar in 1980 and 2010. The magnitude of the MMR remained unchanged in response to adjustment for population change, with the single exception of 1980, when it increased in all-cause and cardiovascular mortality.

**Conclusion:**

The increase in the difference in premature mortality between the richest and poorest areas in Spain is due to the increased difference in cardiovascular mortality. This increase is not accounted for by population change. In rich countries, more empirical evidence is thus needed to test other alternative explanations for the increase in economic differences in mortality.

## Background

Two studies published in the first decade of the present century reported an increase in geographical inequalities in premature mortality in Great Britain from 1991 to 2007 [1,2]. The authors of these studies grouped districts of Great Britain on the basis of an indicator of material wellbeing and found that by 2007 the relative difference in premature mortality in the poorest versus the richest districts had increased with respect to the situation in 1991. These findings confirmed earlier findings obtained by other studies in the United Kingdom (UK) using mortality data from the 1980s. Studies in countries outside the UK, such as the USA and New Zealand [3,4], likewise reported increased differences in mortality between the richest and poorest areas.

While the authors of the above studies attributed their findings to increased differences in wellbeing and income between areas of highest and lowest mortality, they nevertheless failed to show the time trend in differences in the level of material wellbeing between these areas. Indeed, a review of the New Zealand study by Harper raised doubts about whether the trend in inequalities in mortality could be attributed to the increase in economic inequalities [5]. According to Harper, the increase in inequalities in mortality had been observed in a period of declining economic inequalities in New Zealand. Furthermore, he went on to point out that these studies had not always considered the possible influence of other factors, such as differing rates of population growth between some areas and others.

Previously, other authors had already contended that the widening differences in mortality among areas in the UK might be partly attributable to the relationship between population change and mortality [6]. Indeed, some studies conducted in the UK, Sweden and Spain found an inverse relationship between the magnitude of population change and mortality in such areas [7-10]. However, two studies that assessed the trend in geographical differences in premature mortality from 1991 to 2001 in the UK reported different results: while one, based on data from England and Wales, found that the differences in mortality between rich and poor areas were due to the process of migration from some places to others [11], the other, based on Scottish data, found that population change had not contributed to the increase in differences in mortality between rich and poor areas [12].

In order to increase the empirical evidence, this study assesses whether the population change might be responsible for widening differences in mortality between rich and poor areas in the case of a country other than the UK. The reason for specifically choosing Spain was that, during the last third of the 20^th^ and first decade of the 21^st^ centuries, the country had witnessed rising inequalities in mortality between its rich and poor provinces, accompanied by a geographical convergence in income levels and a decrease inequality in per capita income between the poorest and richest provinces [13].

## Methods

Data on the number of deaths by age and cause of death and population data by age were obtained for each province from the National Institute of Statistics (*Instituto Nacional de Estadística*-*INE*) for 1980, 1990, 2000 and 2010. For each of the study years analysed and for each province, age-adjusted premature mortality rates (persons under the age of 75 years) was calculated for all causes and for the following specific causes, i.e., cancer, cardiovascular diseases and external causes, using the standard European population as reference.

The indicator of provincial income used was the per capita gross domestic product (pcGDP) for each of Spain's 50 provinces, calculated as follows: for 1980 and 1990, on the basis of BBVA Foundation estimates of provincial GDP and population [14]; and for 2000 and 2010, on the basis of INE estimates of provincial pcGDP [15]. Similarly, it was calculated the relative increase in the size of the population aged under 75 years in each province over the course of the preceding decade, i.e., for 1980 with respect to 1970, for 1990 with respect to 1980, for 2000 with respect to 1990 and for 2010 with respect to 2000.

In each year analysed, the provinces were grouped into tertiles based on provincial income, and into tertiles based on population change. In each tertile the mean mortality rate was calculated using linear regression, in which the dependent variable was the provincial mortality rate. To this end, dummy variables were previously created so as to be able to classify the tertiles. Similarly, the statistical significance of the linear trend in the mortality rate according to provincial income and population change was assessed.

The magnitude of the association between provincial income and mortality was then assessed as an indicator of inequality in mortality in any given year. The measure of association used was the mortality rate ratio (MRR), with the tertile of provinces with highest provincial income being taken as reference. The logarithm of each province's mortality rate was calculated, and the MRR was obtained via the exponential function of the linear regression coefficient. To ascertain whether the association between provincial income and mortality might be attributable to population change, this latter factor was included in the regression models to check whether the MRR decreased. Due to the fact that provincial density may affect the relationship between provincial income and mortality [16,17], we firstly calculated the MRR according to provincial income, with control for population density.

Furthermore, the study mentioned above raised the possibility that the proportion of the population with a high educational level might have contributed to the increase in differences in mortality by provincial income [13]. Therefore, it was assessed whether the percentage of the population that had completed secondary or higher education in each province might explain the relationship between provincial income and mortality. The data about education, estimated on the basis of general population surveys, were obtained from the Valencian Institute of Economic Research [18].

Finally, in view of the fact that the number of immigrants to Spain rose sharply from 1995 onwards [19], the models used to calculate the MRRs according to provincial income for 2000 and 2010 included the immigrant population's percentage contribution to population change. In general, immigrants show a lower risk of mortality than the native Spanish population for most diseases, but a higher risk of mortality from external causes [20].

## Results

Table 1 shows the premature all-cause mortality rate by provincial income and population change across the study period. Whereas the magnitude of the mortality rate displayed an inverse gradient with provincial income in 2000 and 2010 –i.e., highest magnitude in lowest-income provinces- no relationship was observed in 1980 and 1990. Similarly, the magnitude of the mortality rate displayed a direct gradient with population change in 1980, 1990 and 2000 - i.e., highest magnitude in provinces with greatest population change- but no relationship in 2010.

**Table 1:** **Age-adjusted premature mortality rate for all causes per 100,000 population and 95% confidence interval (CI) according to per capita income and population change of province of residence**

	**1980**				**1990**				**2000**				**2010**			
	**Mortality rate**	**95% CI**			**Mortality rate**	**95% CI**			**Mortality rate**	**95% CI**			**Mortality rate**	**95% CI**		
**Income per capita**																
1st tertile (highest)	428.0	404.5		454.6	360.8	339.2		382.4	288.2	273.8	-	302.7	224.2	213.5	-	234.9
2nd tertile	436.6	401.3	-	470.4	363.4	331.4	-	392.8	313.9	291.2	-	335.4	238.7	223.4	-	255.1
3rd tertile (lowest)	422.4	388.2	-	454.0	380.6	348.3	-	411.7	310.4	287.1	-	331.5	250.2	235.4	-	266.8
p value for trend	0.740				0.208				0.042				<0.001			
**Population change**																
1st tertile (highest)	451.9	430.8		472.9	400.9	382.7		419.1	312.8	297.3		328.3	233.3	221.6		245.1
2nd tertile	434.7	405.6	-	467.6	363.9	338.9	-	391.0	312.0	291.6	-	336.1	235.9	219.5	-	253.6
3rd tertile (lowest)	400.3	372.4	-	428.4	340.0	316.1	-	363.9	285.0	266.5	-	307.1	243.7	227.2	-	262.0
p value for trend	<0.001				<0.001				0.014				0.224			

Spain, 1980, 1990, 2000 and 2010.

Table 2 shows the premature mortality rate by cause of death, according to provincial income and population change. Throughout the study period, the cancer mortality rate showed no association with provincial income: in contrast, the magnitude of the cardiovascular mortality rate displayed an inverse gradient with provincial income in 1990, 2000 and 2010, and the magnitude of the external-cause mortality rate displayed a direct gradient with provincial income in 1980 and 2010. Whereas the magnitude of the cancer and cardiovascular mortality rates displayed a direct gradient with population change in 1980, 1990 and 2000 (though in the lattermost year this gradient was solely observable for cardiovascular mortality), the magnitude of the external-cause mortality rate displayed an inverse gradient in 2010.

**Table 2:** **Premature mortality rate per 100,000 population by cause of death, and 95% confidence interval according to per capita income and population change of province of residence**

	**1980**				**1990**				**2000**				**2010**			
	**MR**	**95% CI**			**MR**	**95% CI**			**MR**	**95% CI**			**MR**	**95% CI**		
**CANCER**																
**Income per capita**																
1st tertile (highest)	119.6	112.3		126.9	125.4	118.1	-	132.8	116.6	111.3	-	121.9	102.4	95.5	-	107.3
2nd tertile	121.7	111.2	-	133.2	121.4	110.8	-	132.1	120.2	112.2	-	128.7	106.5	99.3	-	114.3
3rd tertile (lowest)	109.9	99.9	-	119.3	117.0	106.4	-	126.5	111.8	104.1	-	119.6	103.8	97.0	-	111.3
p value for trend	0.075				0.114				0.298				0.706			
**Population change**																
1st tertile (highest)	124.4	117.6		131.3	128.5	121.6		135.5	115.1	109.7		120.5	101.8	96.9		106.7
2nd tertile	119.4	109.7	-	129.9	120.8	110.9	-	131.1	123.1	115.3	-	131.5	103.6	96.5	-	110.9
3rd tertile (lowest)	107.2	98.1	-	115.9	114.5	105.1	-	124.0	110.7	103.3	-	117.9	107.0	99.5	-	114.1
p value for trend	<0.001				0.007				0.277				0.139			
**Cardiovascular disease**																
**Income per capita**																
1st tertile (highest)	144.3	133.8	-	154.8	90.6	82.7	-	98.4	62.7	56.8	-	68.5	41.3	38.0	-	44.6
2nd tertile	147.9	132.0	-	162.0	95.4	84.1	-	105.8	64.2	65.2	-	82.1	46.6	42.2	-	51.5
3rd tertile (lowest)	150.8	135.5	-	165.8	108.4	96.8	-	121.3	77.7	68.4	-	86.5	54.4	49.0	-	59.6
p value for trend	0.388				0.003				<0.001				<0.001			
**Population change**																
1st tertile (highest)	156.9	147.0		168.9	112.1	105.2		119.0	77.0	70.3		83.6	46.0	41.8	-	50.2
2nd tertile	146.3	133.4	-	162.0	95.9	86.6	-	106.1	72.1	63.7	-	81.6	48.3	42.3	-	54.4
3rd tertile (lowest)	139.8	126.9	-	153.7	86.5	78.3	-	95.7	63.9	57.0	-	73.0	48.0	42.8	-	54.8
p value for trend	0.019				<0.001				0.007				0.503			
**External causes**																
**Income per capita**																
1st tertile (highest)	41.2	37.3		45.0	42.9	39.1		46.7	33.7	30.8		36.7	32.2	28.8		35.5
2nd tertile	41.6	36.2	-	49.2	43.0	37.8	-	48.8	33.4	29.3	-	38.1	34.4	29.6	-	39.7
3rd tertile (lowest)	35.6	30.8	-	41.7	41.2	35.8	-	46.0	31.2	27.4	-	35.8	27.0	23.8	-	31.4
p value for trend	0.044				0.525				0.286				0.047			
**Population change**																
1st tertile (highest)	39.9	35.8		44.0	42.1	38.3		45.9	32.3	29.1		35.4	28.1	25.0		31.2
2nd tertile	38.5	33.2	-	45.9	42.6	37.4	-	48.5	31.3	27.6	-	35.9	28.6	24.9	-	32.9
3rd tertile (lowest)	39.9	34.1	-	46.9	42.3	36.7	-	47.4	35.1	30.7	-	39.9	36.6	31.8	-	41.8
p value for trend	0.994				0.950				0.215				0.005			

Spain, 1980, 1990, 2000 and 2010.

Table 3 shows the premature all-cause MRR according to provincial income, adjusted for different variables in each of the study years. The magnitude of the MRR in the provinces in the poorest versus the richest tertile was 1.01 in 1980 and 1.12 in 2010. The MRR was hardly modified by population change, except in 1980 when its magnitude increased. Adjustment for the percentage of the population that had completed secondary or higher education reduced the magnitude of association of the MRR in 1990, 2000 and 2010. Specifically, the MRRs of provinces in the poorest versus the richest tertile in 1990, 2000 and 2010 were 1.10 (95% confidence interval (CI) 1.02 to 1.20), 1.09 (95% CI 1.02 to 1.17), and 1.12 (95% CI 1.01 to 1.16) respectively, and when adjusted for the variable of education, decreased to 1.07 (95% CI 0.96 to 1.19), 1.04 (95% CI 0.96 to 1.12) and 1.08 (95% CI 1.01 to 1.16) respectively.

**Table 3:** **Premature mortality rate ratio according to per capita income of province of residence adjusted for different variables**

**Tertiles of per capita income**	**1980**		**1990**		**2000**		**2010**	
	**MRR**	**95% CI**	**MRR**	**95% CI**	**MRR**	**95% CI**	**MRR**	**95% CI**
**Model 1 (adjusting for population density)**								
1st tertile (highest)	1.00		1.00		1.00		1.00	
2nd tertile	1.03	0.95-1.12	1.04	0.96-1.12	1.10	1.03-1.18	1.07	1.00-1.14
3rd tertile (lowest)	1.01	0.92-1.10	1.10	1.02-1.20	1.09	1.02-1.17	1.12	1.04-1.19
**Model 2 (Model 1 plus population change)**								
1st tertile (highest)	1.00		1.00		1.00		1.00	
2nd tertile	1.05	0.97-1.13	1.05	0.98-1.13	1.10	1.03-1.18	1.06	0.98-1.13
3rd tertile (lowest)	1.07	0.98-1.17	1.10	1.02-1.18	1.09	1.02-1.16	1.12	1.05-1.20
**Model 3 (Model 1 plus education** ^**1**^ **)**								
1st tertile (highest)	1.00		1.00		1.00		1.00	
2nd tertile	1.03	0.95-1.12	1.04	0.95-1.13	1.07	1.00-1.15	1.07	1.00-1.14
3rd tertile (lowest)	1.00	0.91-1.10	1.07	0.96-1.19	1.04	0.96-1.12	1.08	1.01-1.16

Spain, 1980, 1990, 2000 and 2010.

MRR: Mortality rate ratio.

95% CI: 95% confidence interval.

^1^Percentage of population that had completed secondary or higher education.

Table 4 shows the premature MRRs by cause of death, according to provincial income and adjusted for different variables in each of the study years. In the case of cancer, the MRRs according to provincial income failed to prove significant in any year and their magnitude remained unchanged when adjusted for population change or the percentage of population that had completed secondary or higher education. In cardiovascular mortality, the magnitude of the MRR in the provinces in the poorest versus the richest tertile was 1.08 in 1980 and 1.31 in 2010. The MRR was hardly modified by population change, except in 1980 when its magnitude increased. Adjustment for the variable of education reduced the magnitude of association of the MRR, e.g., in 2010 the MRR in the provinces in the poorest versus the richest tertile went from 1.31 (95% CI 1.18 to 1.44) to 1.26 (95% CI 1.13 to 1.41) when adjusted for education. In the case of mortality due to external causes, the magnitude of the MRR in the provinces in the poorest versus the richest tertile displayed a similar magnitude in 1980 and 2010, namely, 0.80 (95% CI 0.68 to 1.05) and 0.83 (95% CI 0.70-0.95) respectively. This magnitude hardly changed when adjusted for population change or the variable of education.

**Table 4:** **Premature mortality rate ratio by cause of death according to per capita income of province of residence adjusted for different variables**

**Tertiles of per capita income**	**1980**		**1990**		**2000**		**2010**	
	**MRR**	**95% CI**	**MRR**	**95% CI**	**MRR**	**95% CI**	**MRR**	**95% CI**
**Cancer**								
**Model 1 (adjusting for population density)**								
1st tertile (highest)	1.00		1.00		1.00		1.00	
2nd tertile	1.04	0.94-1.14	1.00	0.92-1.09	1.05	0.98-1.12	1.05	0.98-1.13
3rd tertile (lowest)	0.94	0.85-1.03	0.97	0.89-1.06	0.98	0.92-1.05	1.02	0.95-1.09
**Model 2 (Model 1 plus population change)**								
1st tertile (highest)	1.00		1.00		1.00		1.00	
2nd tertile	1.06	0.96-1.16	1.01	0.93-1.09	1.05	0.99-1.11	1.04	0.96-1.12
3rd tertile (lowest)	0.99	0.90-1.10	0.96	0.88-1.05	0.98	0.92-1.04	1.02	0.95-1.10
**Model 3 (Model 1 plus education** ^**1**^ **)**								
1st tertile (highest)	1.00		1.00		1.00		1.00	
2nd tertile	1.05	0.96-1.15	1.01	0.92-1.10	1.05	0.98-1.12	1.05	0.97-1.13
3rd tertile (lowest)	0.96	0.87-1.06	0.98	0.87-1.10	0.98	0.90-1.06	1.01	0.94-1.09
**Cardiovascular disease**								
**Model 1 (adjusting for population density)**								
1st tertile (highest)	1.00		1.00		1.00		1.00	
2nd tertile	1.04	0.94-1.16	1.09	0.98-1.22	1.19	1.06-1.33	1.12	1.01-1.25
3rd tertile (lowest)	1.08	0.97-1.20	1.28	1.14-1.43	1.26	1.12-1.42	1.31	1.18-1.44
**Model 2 (Model 1 plus population change)**								
1st tertile (highest)	1.00		1.00		1.00		1.00	
2nd tertile	1.05	0.95-1.16	1.12	1.03-1.22	1.19	1.07-1.32	1.12	1.00-1.24
3rd tertile (lowest)	1.15	1.03-1.29	1.27	1.16-1.38	1.25	1.12-1.39	1.32	1.20-1.46
**Model 3 (Model 1 plus education** ^**1**^ **)**								
1st tertile (highest)	1.00		1.00		1.00		1.00	
2nd tertile	1.02	0.92-1.14	1.08	0.97-1.21	1.14	1.01-1.27	1.12	1.01-1.24
3rd tertile (lowest)	1.05	0.94-1.17	1.17	1.02-1.36	1.15	1.00-1.31	1.26	1.13-1.41
**External causes**								
**Model 1 (adjusting for population density)**								
1st tertile (highest)	1.00		1.00		1.00		1.00	
2nd tertile	0.97	0.83-1.13	0.98	0.86-1.12	0.97	0.85-1.10	1.02	0.89-1.18
3rd tertile (lowest)	0.80	0.68-0.94	0.92	0.80-1.05	0.90	0.79-1.03	0.83	0.72-0.95
**Model 2 (Model 1 plus population change)**								
1st tertile (highest)	1.00		1.00		1.00		1.00	
2nd tertile	0.95	0.81-1.11	0.98	0.86-1.13	0.97	0.85-1.10	0.95	0.84-1.09
3rd tertile (lowest)	0.78	0.66-0.93	0.92	0.80-1.06	0.90	0.79-1.03	0.83	0.73-0.94
**Model 3 (Model 1 plus education** ^**1**^ **)**								
1st tertile (highest)	1.00		1.00		1.00		1.00	
2nd tertile	0.98	0.83-1.14	0.95	0.83-1.09	0.94	0.82-1.07	1.02	0.88-1.18
3rd tertile (lowest)	0.81	0.69-0.96	0.80	0.67-0.96	0.84	0.72-0.98	0.81	0.69-0.94

Spain, 1980, 1990, 2000 and 2010.

MRR: Mortality rate ratio.

95% CI: 95% confidence interval.

^1^Percentage of population that had completed secondary or higher education.

Adjustment for the contribution of immigrants to population change in 2000 and 2010 failed to modify the MRR estimates for both all-cause mortality and mortality due to the specific causes of death analysed.

## Discussion

### Main findings

The difference in the premature all-cause mortality rate between the poorest and richest provinces in Spain rose from 1980 to 2010; similarly, the difference in the premature cardiovascular mortality rate also increased. In contrast, the relationship between provincial income and cancer and external-cause mortality remained similar throughout the study period. Except for 2010, when no relationship was observed between population change and the premature mortality rate, in 1980, 1990 and 2000 the provinces that experienced the greatest population growth registered the highest premature mortality rates. The above increase in differences between the poorest and richest provinces in terms of all-cause and cardiovascular-disease mortality cannot be attributed to population change.

### Comparison with other studies and possible explanations

This inverse economic gradient in premature all-cause mortality has been observed by studies conducted into premature mortality trends by area of residence in a number of rich countries [1-4,21-23]. Moreover, the economic differences in mortality increased due to the fact that the decline in mortality was greater in the richer and/or less deprived areas. The same was true of our study, in that, from 1980 to 2010, mortality was seen to decline by 48% in the provinces in the richest tertile as opposed to 41% in the provinces in the poorest tertile. The authors of most of the above studies attributed their findings to widening differences in income between the poorest and richest areas. Even so, there has been no empirical evidence to date to support this explanation. Indeed, in Spain the increase in the economic differences in mortality has coincided with a decrease in economic inequalities between rich and poor areas [13].

One possible explanation for this increase in economic differences in mortality, which has been tested empirically, is the relationship between population change and mortality. Among people who move from one place to another the proportion of healthy people is higher than among those who remain in their place of residence. Accordingly, the increase in economic differences in mortality might be due to a greater increase in population in rich than in poor areas. The studies which have tested this explanation have essentially been UK-based, though the results have not been uniform [11,12,24]. And the present study, undertaken in Spain, found that population change did not account for the relationship between provincial income and mortality. Another study conducted in the USA similarly ruled out population change as being responsible for the widening differences in mortality between rich and poor areas [23].

Another important finding in this study is the relationship between population change and mortality, since this finding is contrary to what would have been expected. In 1980, 1990 and 2000 the highest mortality was observed in places with greatest population growth. This finding is in contradiction to previous studies [7-10], where the increased mortality was observed in areas with lower population change. In these previous studies -one of which was undertaken in Spain [10]- small areas such as neighbourhoods, districts and municipalities, were used as the unit of analysis. In the present study, in contrast, provinces were used. Probably the size of the unit of analysis influences the relationship between population change and mortality, since the above-mentioned US study used counties as the unit of analysis and it too failed to observe a relationship between population change and mortality [23].

The present study also assessed the proportion of residents with a high educational level, as an alternative explanation for the increase in economic differences in mortality across time. Given that mortality shows an inverse gradient with educational level [25], then if the percentage of persons with a high educational level were to rise more sharply in rich than in poor areas, any increase in differences in mortality by area income level might well be attributable to this factor. Nevertheless, the findings obtained also rule out this explanation, i.e., although the proportion of subjects with a high educational level would account for a small part of the economic differences in mortality, the increase in such differences remained unchanged in response to adjustment for this variable.

The possible existence of a different immigration pattern depending on the income level of the province of residence was also assessed. The years 2000 and 2010 were analysed, since the rate of immigration to Spain had previously been very low. However, this can also be ruled out as an explanation for the increase in economic differences in mortality, since the results were in no way changed by adjustment for the immigrant population's percentage contribution to population change. Moreover, the increase in the MRR between the poorest and richest provinces was already in evidence between 1980 and 1990, when immigration to Spain was practically non-existent.

Analysis of results by cause of death provides some clues about other alternative explanations. If the MRR results for cancer, cardiovascular diseases and external causes at baseline and at the end of the study period are compared, it will be seen that the findings for both cancer and external-cause mortality do not vary: whereas the cancer MRR in the poorest versus the richest provinces shows no significant differences by provincial income, the external-cause MRR displays a similar magnitude of around 0.80. Similarly, neither population change nor the percentage of subjects with a high educational level alters this relationship between provincial income and mortality due to these two causes of death. In contrast, as happens with all-cause mortality, the cardiovascular-disease MRR in the poorest versus the richest provinces is higher in 2010 than in 1980; and when adjusted for population change and the percentage of the population with a high educational level, the findings are also similar to those obtained for all-cause mortality.

Accordingly, this study's main conclusion is that the increase in economic differences in mortality between rich and poor areas is due to an increase in economic differences in cardiovascular-disease mortality. This increase in differences in cardiovascular-disease mortality by income level of area of residence has also been observed in studies conducted in the USA [2,26]. In Spain, it is unlikely that this increase would be due to an increase in differences in the lethality of these diseases, since this would mean that the effectiveness of the health care system had improved to a greater extent in the richest provinces. Another possible explanation for this finding is an increase in economic differences in the prevalence of cardiovascular disease risk factors: in Spain, the poorest regions have been observed to have a higher frequency of cardiovascular risk factors [27], though there are no studies that have monitored the trend in risk factors in these regions.

### Study strengths and weaknesses

This study adds empirical evidence to the small number of existing studies that have addressed the relationship between population change and mortality, and the possible importance posed by population change to the increase in differences in premature mortality between rich and poor areas.

Some authors have suggested that the relationship between population change and mortality is an artefact because population change reflects area deprivation [28,29]. These studies have been undertaken using small areas as the unit of analysis. Our study enables population change to be ruled out as an indirect indicator of an area's level of deprivation, since, with the single exception of 1980, population change failed to change the relationship with provincial income.

Another of our study's strengths lies in the use of province as the unit of analysis, in view of the availability of death data by age and cause of death at this level of geographical disaggregation. This made it possible to study the change in the relationship between an indicator of area income level and mortality over a period of thirty years, and assess the importance of the role that population change might play in this relationship. The heterogeneity of the findings by cause of death means that cardiovascular-disease risk factors, related to the income level of area of residence can be pinpointed as being responsible for the results observed.

## Conclusion

In summary, the widening difference in premature mortality between the poorest and richest provinces in Spain is due to the increase in the difference in premature mortality due to cardiovascular diseases. This increase is not accounted for by population change in the provinces across the study period. Polarisation of the prevalence of cardiovascular risk factors between areas with highest and lowest income levels might be behind this finding.

## References

[1] Shaw M, Davey Smith G, Dorling D (2005). Health inequalities and New Labour: how the promises compare with real progress. BMJ.

[2] Thomas B, Dorling D, Davey Smith G (2010). Inequalities in premature mortality in Britain: observational study from 1921 to 2007. BMJ.

[3] Singh GK, Siahpush M (2002). Increasing inequalities in all-cause and cardiovascular mortality among US adults aged 25–64 years by area socioeconomic status, 1969–1998. Int J Epidemiol.

[4] Pearce J, Dorling D (2006). Increasing geographical inequalities in health in New Zealand, 1980–2001. Int J Epidemiol.

[5] Harper S (2006). Commentary What explains widening geographic differences in life expectancy in New Zealand. Int J Epidemiol.

[6] Brimblecombe N, Dorling D, Shaw M (2000). Migration and geographical inequalities in health in Britain. Soc Sci Med.

[7] Davey Smith G, Shaw M, Dorling D (1998). Shrinking areas and mortality. Lancet.

[8] Molarius A, Janson S (2000). Population change and mortality in men and women. J Epidemiol Community Health.

[9] Davey Smith G, Shaw M, Dorling D (2001). Population change and mortality in men and women. J Epidemiol Community Health.

[10] Regidor E, Calle ME, Domínguez V, Navarro P (2002). Inequalities in mortality in shrinking and growing areas. J Epidemiol Community Health.

[11] Connolly S, O'Reilly D, Rosato M (2007). Increasing inequalities in health: is it an artefact caused by the selective movement of people?. Soc Sci Med.

[12] Boyle P, Exeter D, Flowerdew R (2004). The role of population change in widening the mortality gap in Scotland. Area.

[13] Regidor E, Santos JM, Ortega P, Calle ME, Astasio P, Martínez D (2014). Decreasing income inequality and emergence of the association between income and premature mortality: Spain, 1970–2010. Health Place.

[14] Fundación BBV (1999). Renta nacional de España y su distribución provincial. Serie homogénea. Años 1955 a 1993 y avances 1994 a 1997.

[15] National Statistical Institute. National Counts. Spanish Regional Accounts. http://www.ine.es/en/inebmenu/mnu_cuentas_en.htm (accessed December 1 2014).

[16] Mahoney MC, LaBrie DS, Nasca PC, Wolfgang PE, Burnett WS (1990). Population density and cancer mortality differentials in New York State, 1978–1982. Int J Epidemiol.

[17] Chaix B, Rosvall M, Lynch J, Merlo J (2006). Disentangling contextual effects on cause-specific mortality in a longitudinal 23-year follow-up study: impact of population density or socioeconomic environment?. Int J Epidemiol.

[18] Valencian Institute of Economic Research. Human Capital. http://www.ivie.es/en/banco/caphum/series.php (accessed December 1, 2014).

[19] Eurostat (2005). Europe in figures. Eurostat Yearbook 2005.

[20] Regidor E, de La Fuente L, Martínez D, Calle ME, Domínguez V (2008). Heterogeneity in cause-specific mortality according to birthplace in immigrant men residing in Madrid, Spain. Ann Epidemiol.

[21] Krieger N, Rehkopf DH, Chen JT, Waternan PD, Marcelli E, Kennedy M (2008). The fall and rise of US inequities in premature mortality: 1960–2002. PLoS Med.

[22] Leyland AH, Dundas R, McLoone P, Boddy FA (2007). Cause-specific inequalities in mortality in Scotland: two decades of change A population-based study. BMC Public Health.

[23] Ezzati M, Friedman AB, Kulkarni SC, Murray CJL (2008). The Reversal of Fortunes: Trends in County Mortality and Cross-County Mortality Disparities in the United States. PLoS Med.

[24] Connolly S, O'Reilly D (2007). The contribution of migration to changes in the distribution of health over time: five-year follow-up study in Northern Ireland. Soc Sci Med.

[25] Reques L, Giráldez-García C, Miqueleiz E, Belza MJ, Regidor E (2014). Educational differences in mortality and the relative importance of different causes of death: a 7-year follow-up study of Spanish adults. J Epidemiol Community Health.

[26] Wing S, Casper M, Riggan W, Hayes C, Tyroler HA (1988). Socioenvironmental characteristics associated with the onset of decline of ischemic heart disease mortality in the United States. Am J Public Health.

[27] Grau M, Elosua R, Cabrera de León A, Guembe MJ, Baena-Díez JM, Alonso T (2011). Cardiovascular risk factors in Spain in the first decade of the 21st century, a pooled analysis with individual data from 11 population-based studies: the DARIOS study. Rev Esp Cardiol.

[28] Exeter DJ, Feng Z, Flowerdew R, Boyle PJ (2005). Shrinking areas and mortality: an artefact of deprivation effects?. J Epidemiol Community Health.

[29] Exeter DJ, Boyle PJ, Feng Z, Boyle M (2009). Shrinking areas and mortality: an artefact of deprivation effects in the West of Scotland?. Health Place.

